# Complete Genome Sequence of an Acetic Acid Bacterium, Acetobacter aceti JCM20276

**DOI:** 10.1128/MRA.00962-20

**Published:** 2020-10-15

**Authors:** Yuu Hirose, Jakkaphan Kumsab, Ryuta Tobe, Hisaaki Mihara

**Affiliations:** a Department of Applied Chemistry and Life Science, Toyohashi University of Technology, Toyohashi, Aichi, Japan; b College of Life Sciences, Ritsumeikan University, Kusatsu, Shiga, Japan; University of Arizona

## Abstract

Acetobacter aceti is used in industry to produce vinegar by converting ethanol into acetic acid. We determined the complete genome sequence of *A*. *aceti* JCM20276, which is composed of one chromosome and four plasmids. This study may contribute to a better understanding of the genes necessary for acetic acid production.

## ANNOUNCEMENT

Acetobacter aceti is an acetic acid bacterium that is industrially important for the production of vinegar (1, 2). To analyze the molecular basis for the glyoxylate metabolism in *A. aceti* JCM20276, formerly known as Gluconobacter dioxyacetonicus IAM1814 (3) (=Asai A15 [4]), we performed whole-genome sequencing of this strain using the MinION (Oxford Nanopore Technologies) and MiSeq (Illumina) platforms. The strain (lyophilized cells) was obtained from the RIKEN BioResource Research Center (BRC) through the National BioResource Project of the Ministry of Education, Culture, Sports, Science and Technology of the Japan Agency for Medical Research and Development (MEXT-AMED). Cells from a single colony were cultured in a lactate medium (0.6% sodium lactate, 0.3% yeast extract, and 0.2% polypeptone). Genomic DNA was purified from lysed cells using a Wizard genomic DNA purification kit (Promega) and further purified using Genomic-tip 20/G (Qiagen). A DNA library for MinION was prepared using a ligation sequencing kit (SQK-LSK108; Oxford Nanopore Technologies) without any size selection of genomic DNA and was sequenced on a MinION device with a nanopore flow cell (R9.4.1; Oxford Nanopore Technologies). Base calling of raw sequence data was performed with MinKNOW v.19.06.8. A total of 28,000 reads (total, 261 Mbp) with a maximum read length of 248 kbp, *N*_50_ read length of 14,291 bp, and *N*_90_ read length of 4,457 bp were obtained from the MinION run, which corresponds to 58× coverage of the *A. aceti* JCM20276 genome. The DNA library for MiSeq was prepared using a KAPA HyperPlus kit (Roche) with 8 cycles of PCR amplification. Each end of 300 bp of the libraries was sequenced on a MiSeq instrument with a MiSeq reagent kit v3 (600 cycles; Illumina). Base calling and demultiplexing of the reads were performed using Real-Time Analysis v1.18.54 and MiSeq Control Software v2.6.21. Low-quality reads were trimmed using fastp v0.20.0 (5). A total of 1.05 million paired reads (total, 535 Mbp) were obtained from the MiSeq run after the trimming, which corresponds to 135× coverage of the *A. aceti* JCM20276 genome. Assembly was performed using Flye v2.7.1 (6) with MinION reads with further polishing using Pilon v1.23 (7) with MiSeq reads with the --fix bases option. Default parameters were used for all software unless otherwise specified. A total of 3,967,915 bp of the *A. aceti* JCM20276 genome was successfully assembled, and it contained a circular chromosome (3,743,357 bp), three circular plasmids (91,838 bp, 84,214 bp, and 2,685 bp), and a putative linear plasmid (45,320 bp). The topology of the circularity of the chromosome and three plasmids was confirmed by checking the presence of the MinION reads spanning both ends of the contigs. A total of 3,788 coding DNA sequences (CDSs), 12 rRNAs, 53 tRNAs, and 3 CRISPR genes were predicted and annotated using DFAST (8). The GC content and coding ratio of the *A. aceti* JCM20276 genome were calculated as 56.6% and 85.1%, respectively. The genome sequence of *A. aceti* JCM20276 will provide new clues to the molecular basis for acetic acid production.

### Data availability.

The complete genome sequence of *A. aceti* JCM20276 was deposited in NCBI/ENA/DDBJ under accession numbers AP023326, AP023327, AP023328, AP023329, and AP023330 (BioProject accession number PRJDB10254 and BioSample accession number SAMD00237440). The raw reads are available under the SRA accession numbers DRX228559 and DRX228560.
